# Analysis of the pathogen spectrum, co-infection characteristics, and risk of severe disease in community-acquired pneumonia among children in Henan province

**DOI:** 10.3389/fped.2026.1873573

**Published:** 2026-07-29

**Authors:** Lu Xiaojing, Li Shufang, Yang Qiuyan, Zhu Zhijie, Zhang Yanli, Xu Qingrong

**Affiliations:** Department of Respiratory Medicine, The Third Affiliated Hospital of Zhengzhou University, Zhengzhou, Henan, China

**Keywords:** children, co-infection, community-acquired pneumonia, pathogen spectrum, severe pneumonia

## Abstract

**Objective:**

To analyze the pathogen spectrum, co-infection characteristics, and risk factors for severe pneumonia in children with community-acquired pneumonia (CAP) in Henan Province.

**Methods:**

This study included 2,149 pediatric inpatients with CAP admitted to the Third Affiliated Hospital of Zhengzhou University from June 2023 to June 2025. Throat swab samples were tested using multiplex polymerase chain reaction (PCR) for 11 respiratory pathogens. Combined with culture results, the pathogen spectrum and co-infection characteristics were analyzed, and risk factors for severe pneumonia were identified using multivariate logistic regression analysis. It should be noted that a positive throat-swab PCR result cannot unambiguously differentiate pathogenic infection from colonization, prior infection, or asymptomatic carriage; this inherent limitation was taken into account during data interpretation.

**Results:**

The overall pathogen detection rate was 89.34%, with *Mycoplasma pneumoniae* (31.55%) and rhinovirus (31.13%) predominating; among bacteria, *Haemophilus influenzae* (3.77%) and *Streptococcus pneumoniae* (3.35%) were the most common. Except for influenza B virus and human coronavirus, significant differences were observed in the age distribution of each pathogen (*P* < 0.05). The detection rate of *M. pneumoniae* increased significantly with age, reaching 58.5% in school-aged children. Rhinovirus was active throughout the year (peaking at 65.14% in October 2024), while *M. pneumoniae* had the highest detection rate in October 2023 (65.93%). The overall co-infection rate was 31.07%, consisting primarily of virus–virus or virus–atypical pathogen combinations (21.49%), with rhinovirus–*M. pneumoniae* being the most common (8.52%). Independent risk factors for severe pneumonia were underlying medical conditions (OR = 4.031) and a history of preterm birth (OR = 3.544), whereas co-infections with viruses or atypical pathogens, or concurrent bacterial infections, did not significantly increase the risk of severe disease.

**Conclusion:**

From June 2023 to June 2025, the pathogen spectrum of CAP in children in Henan Province was dominated by *M. pneumoniae* and rhinovirus (though HRV positivity should be interpreted with caution given its high asymptomatic carriage rate), with a distinct age distribution. Underlying medical conditions and a history of preterm birth were the primary risk factors for severe pneumonia.

## 1 Introduction

Community-acquired pneumonia (CAP) is a leading cause of morbidity and mortality in children, with a particularly heavy burden in low- and middle-income countries (1). The pathogen spectrum of pediatric CAP is complex, exhibiting regional, seasonal, and age-related variations, and primarily includes viruses (respiratory syncytial virus, influenza virus), bacteria (*Streptococcus pneumoniae*), and *Mycoplasma pneumoniae* (2, 3). The incidence of co-infection ranges from approximately 20% to 30%, with higher prevalence observed in younger children (4, 5). However, most large-scale surveillance data predate 2020 and thus fail to fully reflect changes in the epidemiological characteristics of respiratory pathogens following the COVID-19 pandemic. In the post-pandemic era, the prevalence and seasonality of common pathogens have undergone significant alterations (6). Accordingly, this study systematically analyzes the etiological characteristics and co-infection patterns of CAP in children during the post-pandemic era based on clinical data from the Third Affiliated Hospital of Zhengzhou University from June 2023 to June 2025, and explores risk factors for severe pneumonia to provide guidance for clinical diagnosis and treatment.

## 2 Materials and methods

### 2.1 General information

A total of 2,149 pediatric patients with CAP admitted to the Third Affiliated Hospital of Zhengzhou University from June 2023 to June 2025 were included. Inclusion criteria: (1) met the diagnostic criteria of the “Guidelines for the Management of Community-Acquired Pneumonia in Children (2024 Revision)” (7); (2) complete clinical data; (3) age 0–18 years. Exclusion criteria: (1) age >18 years; (2) tuberculosis, pertussis, non-infectious pneumonia, or hospital-acquired pneumonia; (3) incomplete clinical data. Patients were classified as having mild or severe pneumonia according to the guidelines. This study was approved by the Ethics Committee of the Third Affiliated Hospital of Zhengzhou University (Ethics No.: 2024-385-01). Written informed consent was waived for this retrospective study.

### 2.2 Study methods

#### 2.2.1 Clinical data collection

Nasopharyngeal swabs were collected from all pediatric patients on the day of admission. A multiplex respiratory pathogen detection kit (Health Gene Technology, Ningbo, China) based on multiplex polymerase chain reaction (PCR) and capillary electrophoresis fragment analysis (ABI 3500 DX Genetic Analyzer) was used to test for 11 pathogens: influenza A virus (Flu A), influenza B virus (Flu B), human parainfluenza virus (HPIV), adenovirus (ADV), *Chlamydia* *sp.* (CH, note: the assay detects Chlamydia genus-level DNA and cannot distinguish C. pneumoniae from C. trachomatis), human rhinovirus (HRV), human coronavirus (HCoV), human metapneumovirus (HMPV), respiratory syncytial virus (RSV), *Mycoplasma pneumoniae* (MP), and human bocavirus (HBoV). Each reaction incorporated an RNA internal control to monitor nucleic-acid extraction efficiency and inhibition. Every run included a positive control (human genomic DNA spiked with viral RNA fragments) and a negative control (nuclease-free water), as specified in the package insert. A target locus was scored as positive when the peak height exceeded the high standard threshold of the corresponding capillary channel, negative when below the low standard threshold, and equivocal (requiring retest) when falling between the two thresholds. The procedure was strictly performed according to the manufacturer's instructions. Simultaneously, blood, sputum, bronchoalveolar lavage fluid, or pleural effusion samples were collected for bacterial/fungal culture. Patient data including age, gender, symptoms, physical signs, and underlying medical conditions were also collected.

It should be noted that a positive throat swab PCR result may reflect active infection, asymptomatic carriage, colonization, or residual nucleic acid from a prior infection. Therefore, in this retrospective study, pathogen attribution was determined by integrating PCR results with clinical manifestations (fever, cough, abnormal auscultation) and radiological findings. A result was considered etiologically relevant only when the detected pathogen was compatible with the clinical and radiological presentation, and when no other clearly predominant pathogen was present. For atypical pathogens (MP, Chlamydia sp.), pharyngeal swabs were chosen for their minimal invasiveness, good tolerance in children, and routine use at our center, ensuring consistent sampling. They provide sensitivity comparable to nasopharyngeal aspirates for atypical pathogens. Serology was avoided because IgM appears late, a single sample cannot differentiate past from recent infection, and paired testing was retrospectively unfeasible. Thus, swabs offered the most practical approach.

Bacterial or fungal infection was defined as a positive culture result in any specimen (blood, sputum, bronchoalveolar lavage fluid, or pleural effusion) submitted after admission. Underlying conditions included congenital heart disease, respiratory tract malformations, inherited metabolic disorders, neuromuscular diseases, immunodeficiency disorders, anemia, malnutrition, and chronic liver or kidney disease.

### 2.3 Statistical analysis

Statistical analysis was performed using SPSS 25.0 software. Categorical data are presented as frequencies and percentages [*n* (%)]. Intergroup comparisons were conducted using the chi-square test or Fisher's exact test. Multivariate logistic regression analysis was used to identify risk factors for severe pneumonia. The significance level was set at *α* = 0.05.

## 3 Results

### 3.1 Basic characteristics of the study population

**Figure 1:**
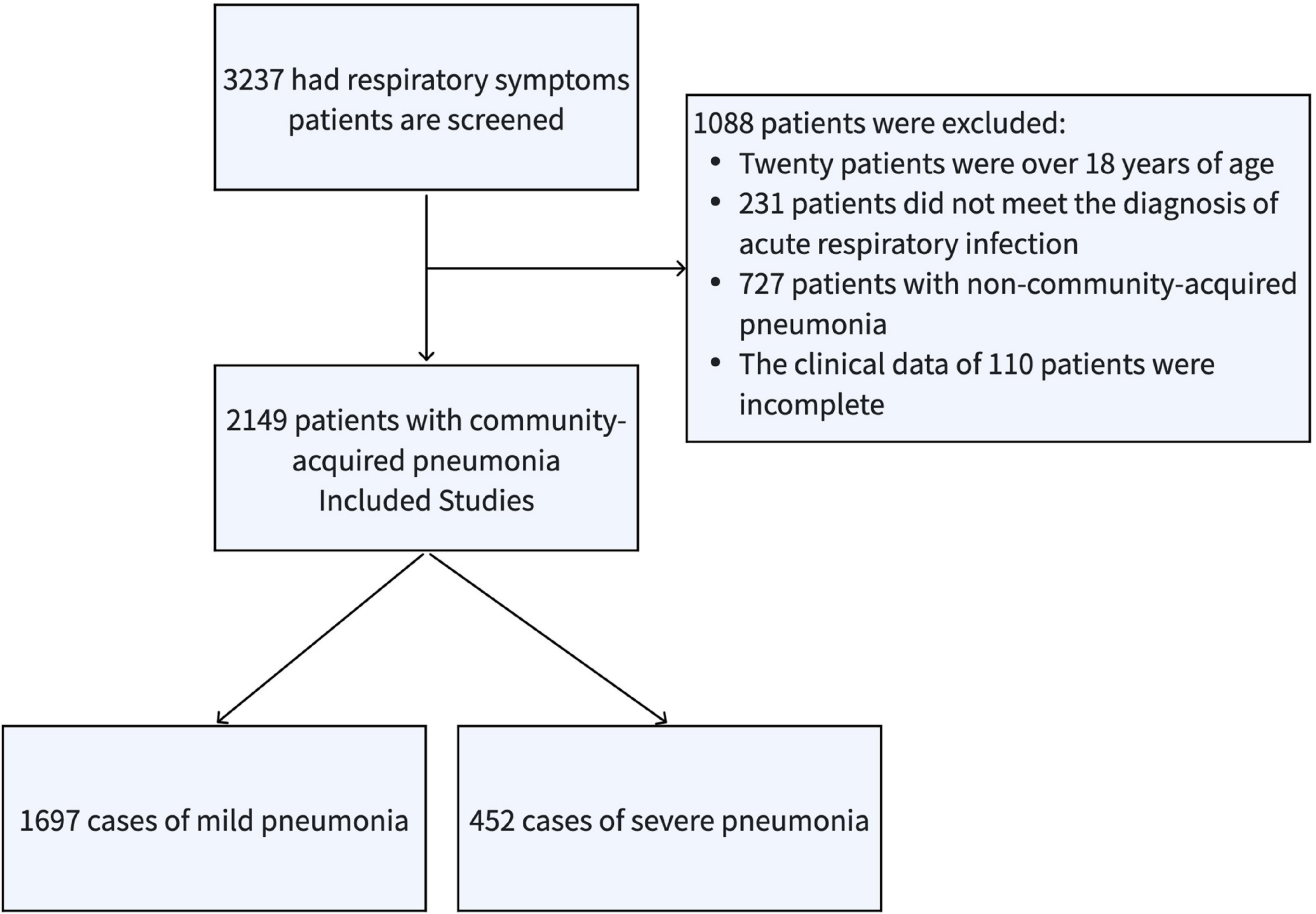
Flow chart of data collection and screening.

A total of 2,149 children with CAP were included, comprising 1,697 cases of mild disease (78.96%) and 452 cases of severe disease (21.04%). The proportion of infants in the severe group was significantly higher than in the mild group, and the prevalence of preterm birth and comorbidities was also significantly higher (*P* < 0.05). The male-to-female ratio was 1.36:1, with no statistically significant difference between groups (see Figure 1 and Table 1). The predominant clinical symptoms were cough (85.2%) and fever (67.4%); wet rales were present in 62.8% of cases, and wheezing in 36.1%.

**Table 1:** Demographic characteristics of children with community-acquired pneumonia [*n* (%)].

Characteristics	Pneumonia (*n* = 2,149)	Mild (*n* = 1,697)	Severe (*n* = 452)	*χ^2^*	*P*-value
**Age group**				47.109	0.000
0–12 months (infancy)	424 (19.73)	289 (17.03)	135 (29.87)		
1–3 years (early childhood)	735 (34.21)	574 (33.82)	161 (35.62)		
4–6 years (preschool age)	564 (26.24)	479 (28.23)	85 (18.80)		
Over 7 years old (school-age)	426 (19.82)	355 (20.92)	71 (15.71)		
**Gender**				0.593	0.441
Female	909 (42.30)	725 (42.72)	184 (40.71)		
Male	1,240 (57.70)	972 (57.27)	268 (59.29)		
**Preterm**				61.471	0.000
No	2,066 (96.14)	1,660 (97.82)	406 (89.82)		
Yes	83 (3.86)	37 (2.18)	46 (10.18)		
**Season**				16.564	0.001
Spring	165 (7.68)	112 (6.60)	53 (11.73)		
Summer	632 (29.41)	490 (28.87)	142 (31.42)		
Fall	1,004 (46.72)	810 (47.73)	194 (42.92)		
Winter	348 (16.19)	285 (16.80)	63 (13.93)		
**Underlying conditions**				73.494	0.000
No	2,044 (95.11)	1,649 (97.17)	395 (87.39)		
Yes	105 (4.89)	48 (2.83)	57 (12.61)		

### 3.2 Detection of respiratory pathogens

**Figure 2:**
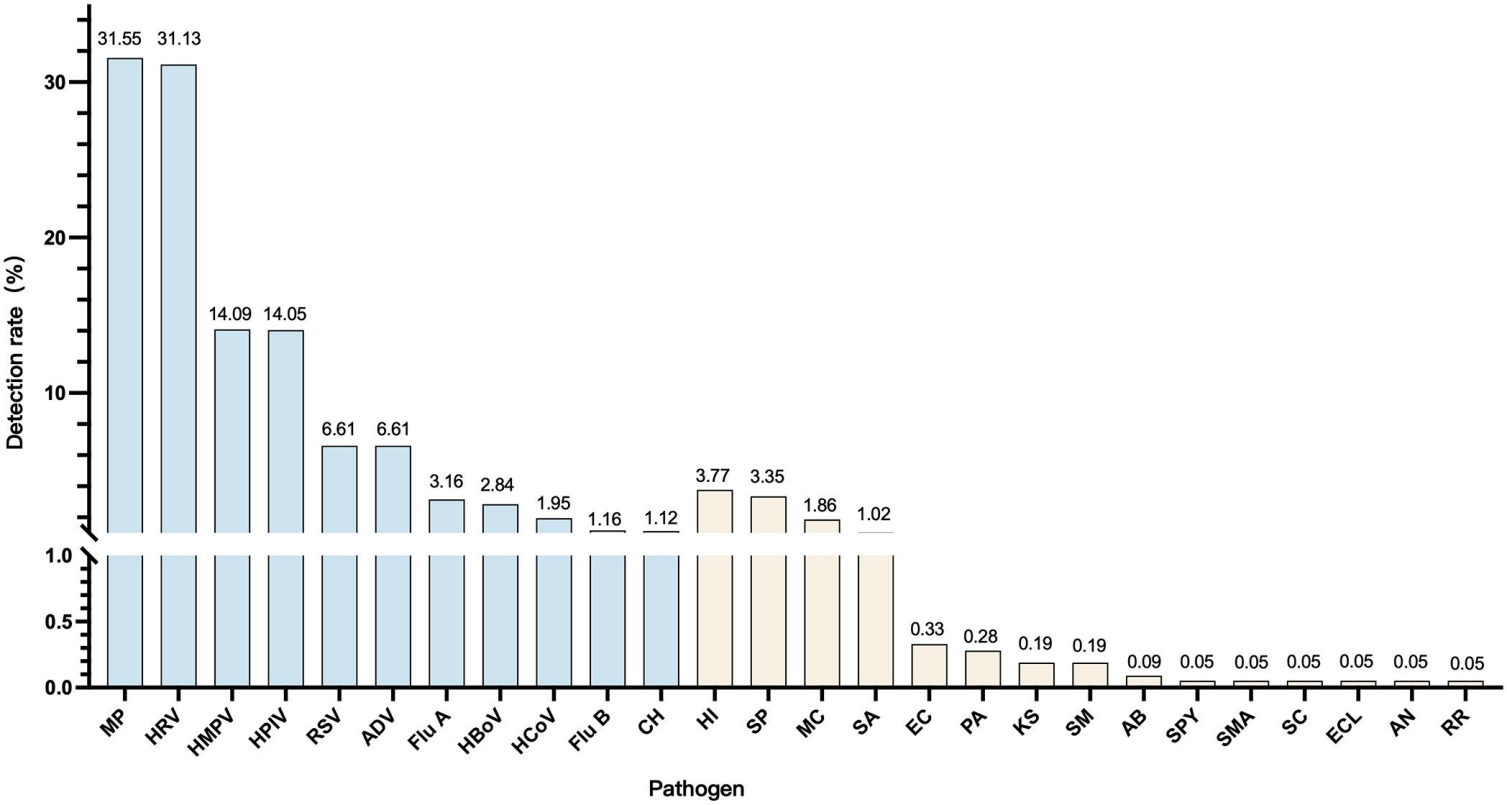
Detection rate of respiratory pathogens in children with community-acquired pneumonia. HI, *Haemophilus influenzae*; SP, *Streptococcus pneumoniae*; MC, *Moraxella catarrhalis*; SA, *Staphylococcus aureus*; EC, *Escherichia coli*; PA, *Pseudomonas aeruginosa*; KS, *Klebsiella*; SM, *Serratia marcescens*; AB, *Acinetobacter baumannii*; SPY, *Streptococcus pyogenes*; SMA, *Stenotrophomonas maltophilia*; SC, *Streptococcus constellatus*; ECL, *Enterobacter cloacae*; AN, *Aspergillus Niger*; RR, *Rhizopus* sp.

The overall pathogen detection rate was 89.34%, with a single-pathogen infection rate of 56.87% (1,222 cases), and a co-infection rate of 31.07% (668 cases), including 25.59% (550 cases) dual infections, 4.51% (97 cases) triple infections, 0.93% (20 cases) quadruple infections, and 0.04% (1 case) quintuple infection. MP had the highest detection rate (31.55%), followed by HRV, HMPV, and HPIV. The detection rate for bacterial/fungal cultures was 10.84% (233 cases), with *Haemophilus influenzae* and *Streptococcus pneumoniae* being the primary bacterial pathogens; fungi and opportunistic pathogens were detected very rarely. See Figure 2.

### 3.3 Age distribution characteristics of pathogens

**Figure 3:**
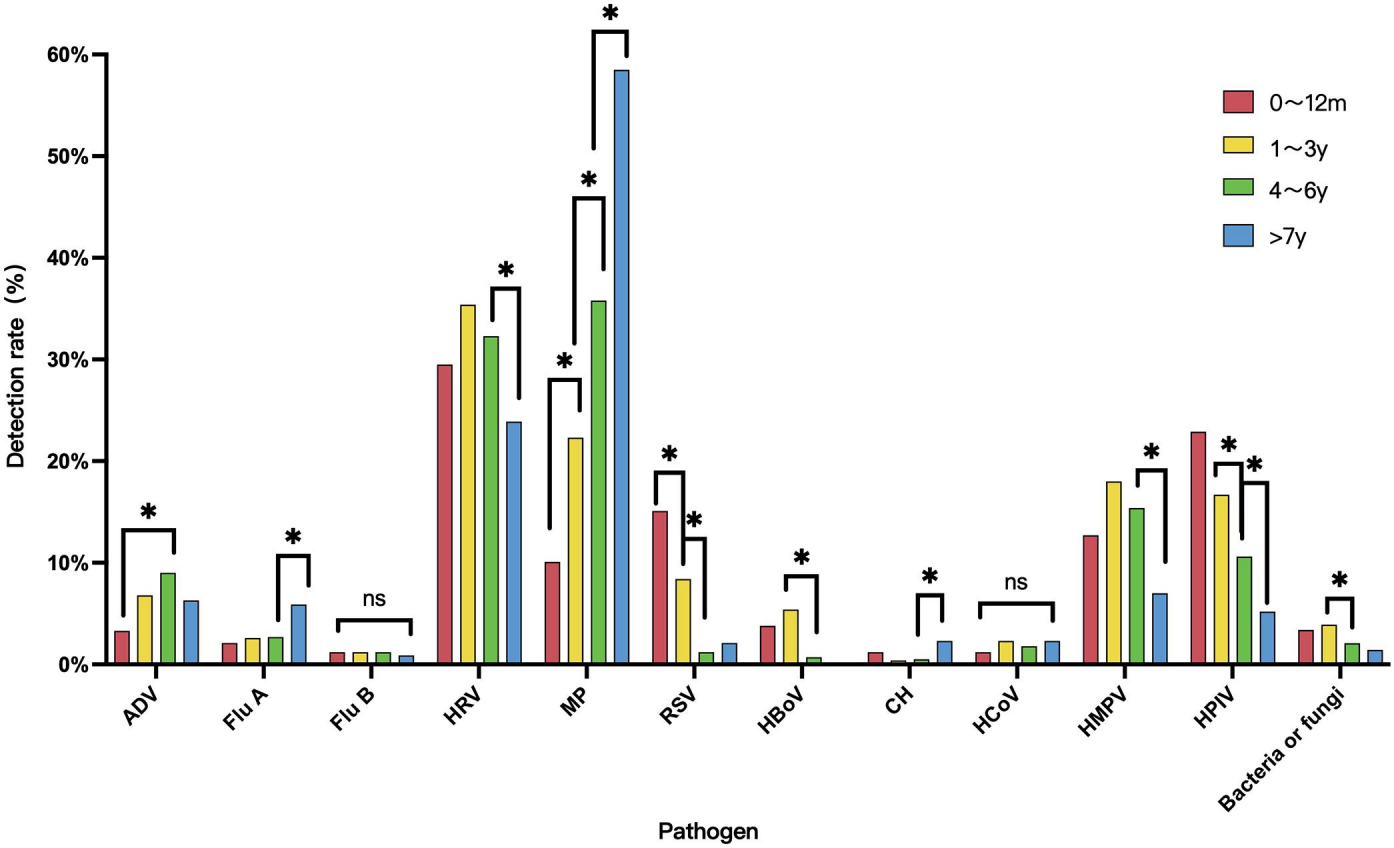
Detection rate of respiratory pathogens in children with community-acquired pneumonia in different age groups. *Indicates a statistically significant difference in detection rates between age groups (*P* < 0.05).

The overall pathogen detection rates for infants, toddlers, preschoolers, and school-aged children were 82.78%, 89.93%, 88.47%, and 88.96%, respectively, with the infant group showing a significantly lower rate than the other groups (*P* < 0.05). Except for influenza B virus and HCoV, significant differences in the age distribution of pathogens were observed (*P* < 0.05). The infant group was dominated by HRV and HPIV, with RSV and HMPV also prominent. Compared to the infant group, the detection rates of HRV and MP increased in the toddler group, while HPIV and HMPV remained at high levels. The detection rate of MP increased significantly with age, reaching 58.5% in the school-age group, where it became the predominant pathogen. Flu A and *Chlamydia sp.* were also more common in the school-age group. Detection rates of HPIV, HMPV, and RSV decreased in the school-age group. In the infant group, the Chlamydia sp. signal may partly reflect C. trachomatis rather than C. pneumoniae, because the assay detects Chlamydia genus-level DNA without species discrimination. Bacterial/fungal detection rates were slightly higher in children under 3 years of age, with a small peak observed in the preschool group. See Figure 3.

### 3.4 Temporal distribution characteristics of pathogens

**Figure 4:**
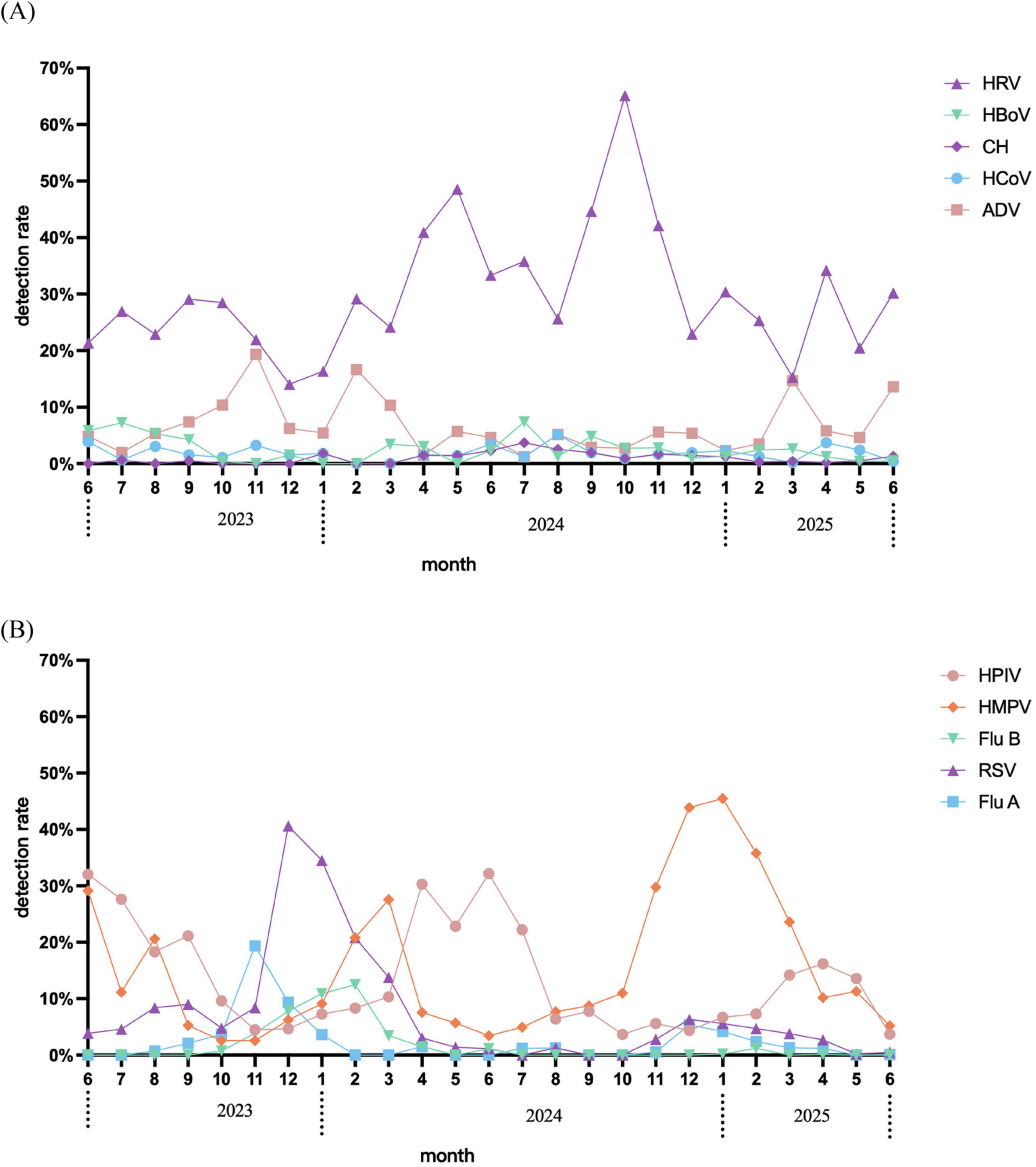
Monthly distribution and detection rate of 11 respiratory pathogens in throat swabs. **(A,B)** Show individual pathogen trends.

All pathogen types exhibited distinct seasonal fluctuations. Influenza A peaked in November 2023, while influenza B reached its highest detection rate in February 2024. HRV remained active throughout the year, reaching as high as 65.14% in October 2024; MP had its highest detection rate in October 2023, after which it gradually declined. RSV peaked in December 2023. ADV showed a minor peak in November 2023, while HPIV reached 32.04% in June 2023. The outbreak of HMPV occurred in late 2024 and early 2025. Detection rates for HBoV, HCoV, and *Chlamydia sp.* were generally low, with mostly sporadic cases. See Figure 4.

### 3.5 Co-infection patterns and common combinations

**Figure 5:**
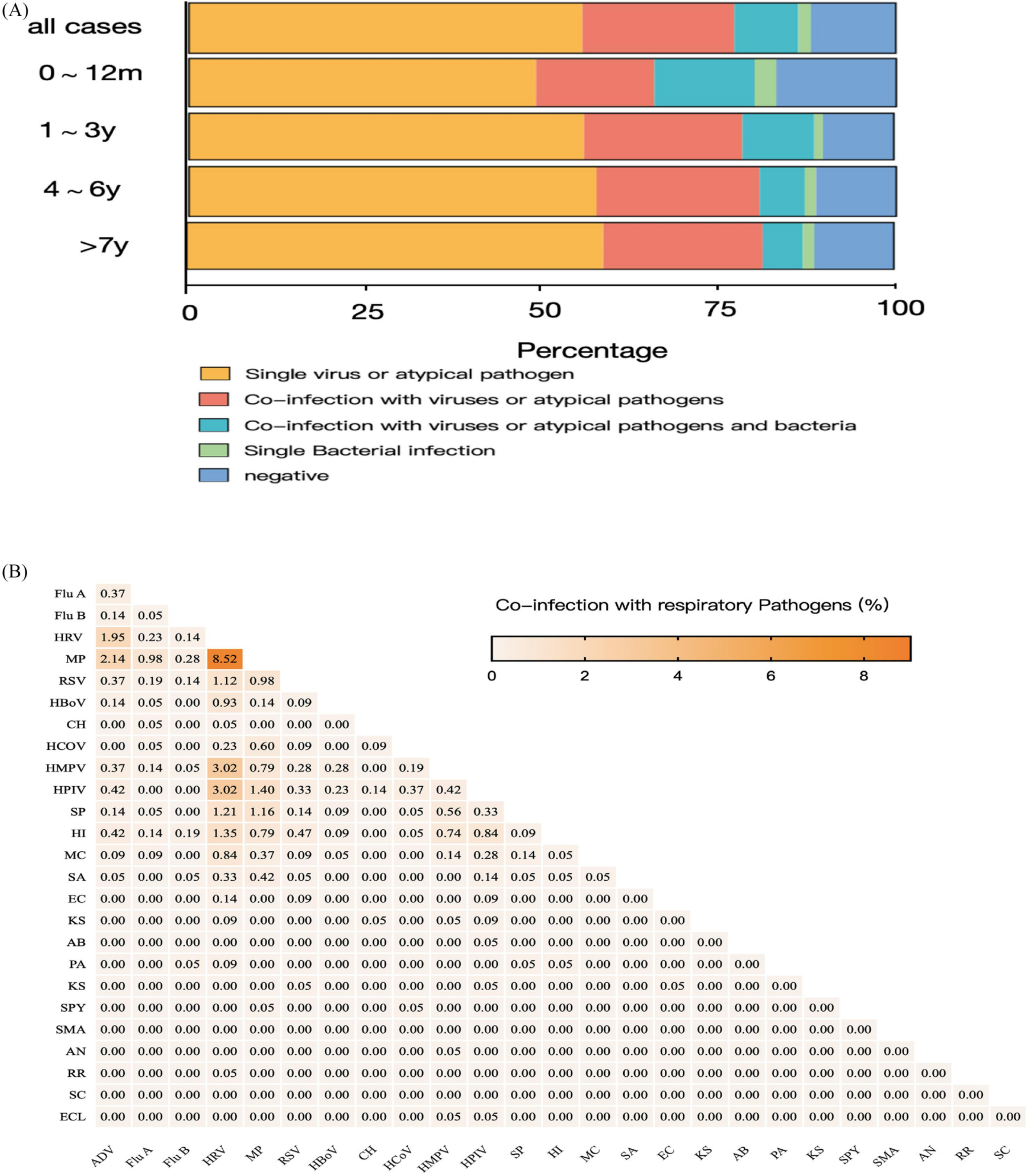
Age-specific co-infections in children with community-acquired pneumonia. **(A)** Age-specific proportions of infection and co-infection patterns. **(B)** Heatmap of respiratory pathogen co-infection rates. HI, *Haemophilus influenzae*; SP, *Streptococcus pneumoniae*; MC, *Moraxella catarrhalis*; SA, *Staphylococcus aureus*; EC, *Escherichia coli*; PA, *Pseudomonas aeruginosa*; KS, *Klebsiella*; SM, *Serratia marcescens*; AB, *Acinetobacter baumannii*; SPY, *Streptococcus pyogenes*; SMA, *Stenotrophomonas maltophilia*; SC, *Streptococcus constellatus*; ECL, *Enterobacter cloacae*; AN, *Aspergillus Niger*; RR, *Rhizopus* sp.

The overall co-infection rates were similar across all age groups (31.36%–36.45%), with no statistically significant differences (*P* > 0.05). The overall co-infection rate was 31.07%, of which 21.49% were virus/atypical pathogen co-infections and 9.07% were virus/atypical pathogen–bacterial co-infections. Infection patterns varied by age group: the infant group had the highest rate of virus/atypical pathogen–bacterial co-infections (14.17%), while the other three groups were dominated by virus/atypical pathogen co-infections (22.44%–23.05%). Among common co-infection combinations, HRV–MP was the most frequent, followed by HRV–HPIV and HRV–HMPV. Among viral–bacterial co-infections, HRV–*H. influenzae* and HRV–*S. pneumoniae* were the most common, followed by MP–*S. pneumoniae*. See Figure 5.

### 3.6 Analysis of risk factors for severe pneumonia

**Figure 6:**
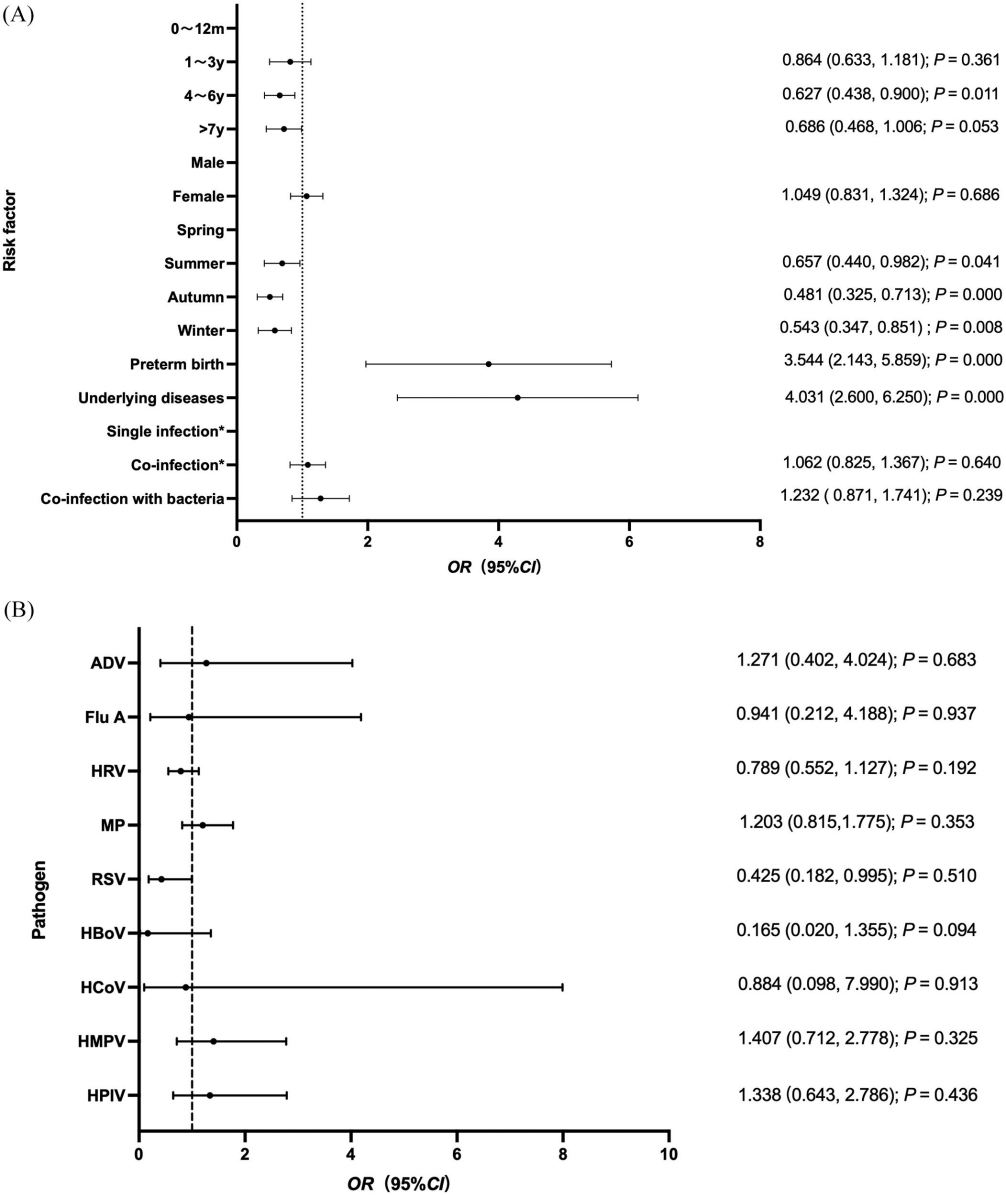
Risk factors for severe community-acquired pneumonia in children. **(A)** Multivariate logistic regression analysis of risk factors for severe pneumonia. **(B)** Comparison of co-infections with viruses or atypical pathogens vs. single infections. *Refers to single infection and co-infection with viruses or atypical pathogens.

Among 1,858 children with at least one positive throat swab test, 416 (22.39%) had severe pneumonia. Multivariate logistic regression with severe pneumonia as the dependent variable and age-stratified analysis revealed that the 4–6-year-old group served as a protective factor compared to the 0–12-month-old group, while underlying diseases and a history of preterm birth were independent risk factors for severe pneumonia. Co-infection with viruses and atypical pathogens, as well as concurrent bacterial infection, did not significantly increase the risk of severe pneumonia. Further multivariate logistic regression analysis comparing co-infection with specific pathogens and other pathogens to single-pathogen infection showed that neither significantly increased the risk of severe pneumonia. Due to the small sample size, we did not conduct an analysis comparing co-infection with influenza B and *Chlamydia sp.* to single-pathogen infection. See Figure 6.

## 4 Discussion

The pathogen spectrum of pediatric CAP is complex, and the epidemiological characteristics of pathogens have undergone significant changes in the post-pandemic era. By analyzing data from pediatric CAP patients hospitalized between June 2023 and June 2025, this study systematically elucidated the pathogen spectrum, co-infection patterns, and risk factors for severe disease in post-pandemic pediatric respiratory infections. These findings provide a reference for precise clinical diagnosis and treatment and facilitate enhanced early identification and intervention for children with high-risk host factors.

The overall pathogen detection rate in this study reached 89.34%, which is comparable to data from international studies (5), highlighting the significant value of microbiological testing in the diagnosis and treatment of pediatric CAP. MP emerged as the most common pathogen (31.55%), consistent with reports of MP outbreaks in China following the pandemic (8). Its detection rate increased significantly with age, reaching 58.5% in the group over 7 years old, consistent with the age distribution characteristics of MP infection. The epidemic peak at the end of 2023 may be related to MP's inherent 3–5-year epidemic cycle and the “immunological debt” following the lifting of non-pharmaceutical interventions (9). HRV was the second most common pathogen (31.13%), similar to the 22.1% reported by Pratt et al. (10), confirming its significant role in pediatric CAP. Its high prevalence is associated with diverse transmission routes and strong environmental resistance. HRV is active year-round, with peaks in spring and autumn. However, Audi et al. (11) noted that HRV circulates year-round in temperate regions, with occasional peaks in autumn and winter. It should be noted that HRV shedding can last up to 2 weeks, and its detection rate in the upper respiratory tract of asymptomatic children reaches 17.3% (12); therefore, positive throat swab results should be interpreted with caution, and future studies should compare upper and lower respiratory tract samples to clarify its pathogenic role.

The detection rate of HMPV was 14.09%, higher than the 6.5% reported by Pratt et al. (10), but consistent with reports of an HMPV outbreak in China beginning in late 2024 and early 2025 (13), which is thought to be related to a resurgence of the pathogen following the lifting of non-pharmaceutical interventions. The detection rate of RSV (6.61%) was significantly lower than the 28% reported in U.S. studies (5), which may be related to the fact that some children clinically suspected of having RSV infection at our center underwent rapid antigen testing rather than PCR testing. The detection rate of CH was extremely low, but showed a slight rebound in the group over 7 years of age. Similar to *M. pneumoniae*, *Chlamydia pneumoniae* is also highly prevalent among school-aged children. However, the assay kit used in this study could only detect *Chlamydia* and could not distinguish between *C. pneumoniae* and *Chlamydia trachomatis*; therefore, the increased detection rate in the infant group may be attributed to *C. trachomatis*.

Bacterial pathogens were primarily *Haemophilus influenzae* and *Streptococcus pneumoniae*, consistent with previous literature reports (14). However, the culture positivity rate was only 10.84%, reflecting the limitations of culture methods in terms of sensitivity. The overall detection rate of bacterial and fungal pathogens was low and decreased with age, with a small peak (3.91%) observed in the 1–3-year-old group. This may be related to the waning of maternal antibodies and the immaturity of the immune system. This suggests that empirical antimicrobial therapy in infancy and early childhood should still cover common community-acquired pathogens such as *S. pneumoniae* and *H. influenzae*.

This study showed a pathogen co-infection rate of 31.07%, slightly higher than the 26% reported in the United States (5), which may be related to the co-circulation of multiple pathogens following the pandemic and “immune debt.” Co-infection patterns exhibited age-specific heterogeneity: the infant group had the highest rate of virus–bacteria co-infection (14.17%), consistent with the immaturity of their immune systems and respiratory barrier function; while older children primarily exhibited co-infections involving viruses and atypical pathogens. This study found that co-infections were predominantly virus–virus or virus–atypical pathogen combinations, with co-infection rates for HRV with MP, HMPV, and HPIV (8.52%, 3.02%, and 3.02%, respectively) being significantly higher than other combinations, similar to the highest co-infection rates reported for MP and HRV in Shanghai following the pandemic (15). In contrast, the rates of viral–bacterial co-infections were generally low, primarily involving *H. influenzae* with HPIV and HMPV, as well as *S. pneumoniae* with HRV. The pathophysiology of virus–bacteria co-infections is multifactorial and relates to anatomical changes in the lungs that allow bacterial invasion and infection (16). Although the overall rate of bacterial co-infection is not high, healthcare providers should remain vigilant for the possibility of secondary bacterial infections in children infected with HPIV or HMPV, particularly in infants and young children.

Logistic regression analysis indicated that the presence of underlying medical conditions (OR = 4.031) and a history of preterm birth (OR = 3.544) were independent risk factors for severe pneumonia, consistent with findings from domestic and international studies (17, 18). Children with underlying medical conditions often have impaired immune defenses, while preterm infants suffer from immature lung development and immune system deficiencies, making them more susceptible to developing severe disease. It is worth further exploration that in this study, various forms of co-infection (including viral–bacterial co-infection) did not significantly increase the risk of severe pneumonia. This may imply that, in determining disease severity, the child's host susceptibility and immune response status are more critical than the quantity of pathogens. However, an important caveat must be acknowledged: the bacterial and fungal culture positivity rate in this cohort was only 10.84%, which almost certainly underestimates the true prevalence of concurrent bacterial infections. Cultures obtained from sputum and nasopharyngeal specimens are subject to contamination and variable pre-analytic conditions, and prior antibiotic treatment—common in hospitalized children—further reduces sensitivity. Consequently, a substantial number of bacterial co-infections may have been missed, which could have biased the regression analysis toward a null finding for bacterial co-infection as a risk factor for severity. This conclusion should therefore be interpreted with caution, and future prospective studies employing more sensitive bacterial detection methods (e.g., metagenomic next-generation sequencing, urinary antigen testing, or multiplex PCR for bacterial targets) are needed to re-examine this association. Currently, the relationship between clinical severity and single or multiple respiratory pathogens remains unclear. Asner et al. (19) observed comparable clinical severity in children with single-virus infections and viral co-infections; a meta-analysis by Lim et al. (20) also indicated that viral co-infections in children were not associated with increased clinical severity, emphasizing that baseline health status is central to assessing disease severity. However, other studies have shown that co-infections are associated with higher intensive care unit (ICU) admission rates, mechanical ventilation rates, and longer ICU stays (21–23). Deol et al. (24) noted that clinical outcomes of respiratory viral co-infections do not follow a single pattern. Differences in study results may stem from variations in patient populations, specific viral types, or study designs. The increased disease severity associated with co-infections may be limited to specific viral combinations; future large-scale studies are needed to explore this by comparing the severity and prognosis of CAP patients based on different pathogen combinations.

This study has several limitations: First, as a single-center retrospective study, caution is warranted when extrapolating results to the national population; second, inclusion was limited to hospitalized children, which may introduce selection bias toward severe cases; third, a positive throat swab PCR result may indicate colonization, asymptomatic infection, or past infection and does not necessarily represent the pathogen causing the current pneumonia. Fourth, the bacterial culture positivity rate of 10.84% likely underestimates true bacterial co-infection prevalence; the conclusion that co-infections do not increase severe-disease risk should be interpreted accordingly and confirmed with more sensitive methods in future work. Fifth, the assay cannot distinguish C. pneumoniae from C. trachomatis; species-level Chlamydia data should be interpreted with this constraint in mind. Sixth, throat swabs—rather than nasopharyngeal aspirates or paired serology—were used to detect atypical pathogens (MP, Chlamydia sp.); this may have reduced analytical sensitivity for these organisms.

In this retrospective study, to differentiate active disease from passive shedding, our clinical team adopted the following criteria during data collection and interpretation: (1) the presence of compatible clinical symptoms (e.g., fever, cough, tachypnea, and auscultatory findings) concurrent with the positive test result; (2) radiological confirmation of pulmonary infiltration or consolidation; and (3) for viruses with high asymptomatic carriage rates (e.g., HRV), we interpreted the result as causative only if no other more definitive pathogen (e.g., MP with higher load) was clearly predominant, or if the clinical presentation matched the typical profile of that virus. If a positive result occurred without corresponding clinical or radiological signs, or in the presence of a clearly dominant alternative pathogen, we considered it colonization/passive shedding rather than the primary etiology.

The findings of this study indicate that clinicians should prioritize age-based pathogen assessment and strengthen early identification and intervention for children with underlying conditions or a history of preterm birth to achieve precise diagnosis and treatment. These descriptive findings lay the foundation for hypothesis-driven research on pathogen–host and pathogen–pathogen interactions. Future longitudinal, immunological, and molecular studies are necessary to further explore pathogenic interaction mechanisms and guide personalized and population-level interventions for CAP.

## Data Availability

The de-identified data may be made available to qualified researchers upon reasonable written request to the corresponding author, subject to ethics committee approval.
